# Remarks upon Vineyards

**Published:** 1860

**Authors:** 


### ARTICLE IV.

*Remarks upon Vineyards.* By an "OBSERVER."

There is an evil in our land, over which Piety has prayed and Philanthropy wept, which I greatly fear is to be wonderfully increased by the mistaken zeal of those who wish its extirpation. Without enlargement, simply to say that Intemperance is a curse by the side of which, War, Famine and Pestilence "pale their ineffectual fires," is to briefly express what every reasoning individual knows.

Oh! it is a sad sight to see so many brave barks go down beneath its treacherous waters; to know that Legislation is licensing a foe to spread death in the ranks of those over whom it should extend its protection; to feel that the friend of your bosom is being bound by chains festooned in roses, and that over all our prosperity a Demon "holds high State," at the waving of whose wand a blight will fall as destructive as frost to summer buds!

One great object gained by the Temperance reformation was the correct ideas it infused in the mind of the people.—Before that, men thought that by alcohol the sick were made well, the well made better. If one were wet, take a drink; if dry, take a drink; if hungry, take a drink to stay the stomach; if filled to repletion, take a drink to facilitate digestion; if unhappy, take a drink to cheer the heart; if pleasure were "tingling the blood" and thrilling the nerves, still, take a drink; if warm, if cold, if busy, if idle, under any circumstances, fly to the panacea—a drink.

A little reasoning exposed the fallacy which for years had eclipsed the truth. Alcohol could not be what its advocates claimed, as, then, it must produce diametrically opposite results. It could not be serviceable in hot weather, as its mediate effect was to debilitate; nor in cold weather, as the unbroken experience of those who have endured the rigors of an Arctic winter, assert; nor to a well man, since those who use it not at all are less subject to disease than those who indulge in it habitually; nor to one sick, only as a poison might be administered as one would laudanum or prussic acid—not so soon, it is true, bringing swift destruction, but yet the more to be dreaded, as the sapper and miner, than the brave foe who meets you face to face with his cry of defiance ringing from his lips.

All intelligent physicians will bear witness to the taste for ardent spirits created in sickness by the tender assiduity of the Doctor who recommended a little wine, and for that reason are cautious in prescribing it when a substitute is at hand. There is no good reason why alcohol, in any form, should be used by one in good health. In the system an enemy encroaching more and more with each succeeding day, until at last, from conquering the outposts, it captures the citadel.—I converse with highly intelligent gentlemen—Temperance advocates too—who think some remedy for the evil I have been portraying, is to be found in the culture of the grape.—I beg leave to differ from them *toto cœlo.* I believe that no reason can be given why, if our rivers should blush in wine, that intemperance would lose many, if any, of its victims.—If wine were hawked about our streets at a nominal price, instead of making the number of drunkards less, it would multitudinously increase them.

I believe that if these old red hills of Georgia, whose soil has been borne away by the neighboring streams, should bear the trailing vine, reeling with clusters of grape, purple and gushing, that a day would arrive when alcohol would be lord of all, and we would be a nation of wine-bibbing drunkards. Suppose wine as abundant as it is in some portions of Europe, would that occasion the non-use of the more ardent kinds of spirits? If this question be answered in the affirmative, then two other questions present themselves. Would the wine here made be any the less adulterated than are whisky, brandy, gin, &c.? If not, would the effect resulting be any the less disastrous than that which now deluges the land? To the first question I answer no, and unless it can be shown then that the use of wine in itself is a positive blessing, I have nothing more to do but to make that answer good by argument. If the manufacture of wine will not cure the drunkard, nor be a positive good to others, I cannot conceive its utility, unless it may *prevent* drunkeness.

Drunkeness is a disease manifested in an inordinate desire for, and a corresponding indulgence in the stimulus of alcohol; how, or why, we may not be able to tell, but the indulgence of any stimulus creates an artificial physical necessity, which will be ministered to or not, as the victim has opportunity, connected with resisting power on his part. Alcohol induces a certain state. A drunkard will use enough for that purpose, whatever it be to produce that particular effect.—He will drink until that condition is produced, whether it be whisky rectified, or wine as weak as it is to be found. *Drunk must come*, whether it takes a half-pint of brandy, or a quart of anything else not so strong.

If wine and strong liquors both were easy of access, the drunkard would use the strong liquors because *drunk would come sooner and with less trouble.* The only way to induce him to drink wine as a substitute, would be to render impossible the obtainment of that which has been not inaptly termed, "*the common drink of the country.*" So long as upper Georgia and Tennessee and other States make corn as easy as they do now, so long will whiskey be distilled; and so long as bees are busy and peach-trees bloom, so long will honey and peach brandy be mixed. Wine, then, even if as pure as the nectar of the gods, will not save the drunkard.

Whom, then, will it benefit? In the first place, I believe that wine in this country—the proportion of agriculture and other business remaining as at present—would never be made so abundantly but that it would be drugged, if it were the interest of the producer; and that it would be to his interest, I do not doubt; since, if he will adulterate it, he can produce twice as much intoxication with the same wine. Suppose it poisoned as other liquors now are, and almost every body induced to indulge in it by the Siren cry, "No danger!" how much better would we be than if the same number of persons were habitually using Alcohol as it is now sold? A nation of drugged wine drunkards are in as sad a plight as a nation of drugged whiskey drunkards, and I would as soon see this land filled with the one as the other, and would sooner see thirty thousand die annually from mean brandy, than thirty millions under the daily use of wine, which it was the interest of the producer to adulterate, and which would therefore receive all the drugs which the diabolical ingenuity of Avarice might deem it policy to add.

It is said that wine is drank in its pure state in Europe by all classes. I doubt it. At all events, the European wines we obtain are sadly changed since they left home, if they were not altered previously. A slight glance at the character of our people and their occupations, will show that, however pure wines may be in foreign countries, our domestic wines would not rival them in native purity and cheapness.

The South raises cotton. Dickens says truly, "the fate of the commercial world literally hangs on a thread." Would the South transfer her laborers from clothing the world to making grapes? Would it be to her pecuniary advantage? According to our population, it is absurd to suppose that we would ever make wine as they do in the old world.

We wield the rod of commercial empire now. Alexander conquered the world, yet wine was his master. Let us not uncrown the South and place the diadem on the head of Bacchus.

I take the broad ground, that wine, even in its purity and as abundant as our never-failing streams, is a "mocker"; an enemy to be dreaded more than whisky or brandy, as it presents a more seductive phase than they. It is the river which empties into the sea of intemperance, and will assuredly carry there its victim, if he surrenders himself to its current. Wine it its *pure* state is a *poison*. It contains alcohol, and its effect on the system is injurious.

If the culture of the grape, then, would save all those who are whisky drunkards, and prevent any more from becoming such, and, yet, would deliver all our people to what the Bible calls a "mocker," what would we gain? It would be like burning a house to get rid of the rats. It would be making of the worth of the country a scape-goat whereby the sin of whisky would be carried into the wilderness. From the bones of wine's victims, a ladder would be made by which to rescue the drunkard from the malebolge in which his passions had precipitated him. There is no safety but in abstinence. Wine implants a taste for a stimulus, which alone the fieriest draughts can quench. The safe side is, to touch not, handle not, taste not. It "is a mocker." Within its rosy depths how many a bright light has been extinguished! On its surface, smooth as summer's sea and phosphorescent with its flashiug light, there lurks the monster and the rock, and you who dread the shipwreck and the maelstroom, embark not, I pray you, on its tempting wave. Marion, when in

South Carolina, men were slow to give up their gains and risk all for liberty, heard with satisfaction, melancholy it is true, when British tyranny had burned some Royalist's home, and wasted some Conservative's substance. And why? Because he wished that men should thereby have their eyes opened to the wrongs under which the Colonies were suffering, and against which he was struggling. And so, when I hear that whisky has called to its aid the virulence of other poison, I, too, have not an unmingled regret, since thereby men may know the character of the foe, intemperance, with which we have to contend.

